# Real-Time Measurements and Characterization of Airborne Particulate Matter from a Primary Silicon Carbide Production Plant

**DOI:** 10.3390/ijerph14121611

**Published:** 2017-12-20

**Authors:** Rikke Bramming Jørgensen, Ida Teresia Kero

**Affiliations:** 1 Department of Industrial Economics and Technology Management, Norwegian University of Science and Technology, NO-7491 Trondheim, Norway; 2 Department of Industrial Process Technology, SINTEF Materials and Chemistry, P.O. Box. 4760, NO-7465 Trondheim, Norway; ida.kero@sintef.no

**Keywords:** SiC, UFP, Acheson process, FMPS, ELPI, CPC, nanotechnology, ultrafine particles, workplace

## Abstract

Airborne particulate matter in the silicon carbide (SiC) industry is a known health hazard. The aims of this study were to elucidate whether the particulate matter generated inside the Acheson furnace during active operation is representative of the overall particulate matter in the furnace hall, and whether the Acheson furnaces are the main sources of ultrafine particles (UFP) in primary SiC production. The number concentration of ultrafine particles was evaluated using an Electrical Low Pressure Impactor (ELPI^TM^, Dekati Ltd., Tampere, Finland), a Fast Mobility Particle Sizer (FMPS^TM^, TSI, Shoreview, MN, USA) and a Condensation Particle Counter (CPC, TSI, Shoreview, MN, USA). The results are discussed in terms of particle number concentration, particle size distribution and are also characterized by means of electron microscopy (TEM/SEM). Two locations were investigated; the industrial Acheson process furnace hall and a pilot furnace hall; both of which represent an active operating furnace. The geometric mean of the particle number concentration in the Acheson process furnace hall was 7.7 × 10^4^ particles/cm^3^ for the UFP fraction and 1.0 × 10^5^ particles/cm^3^ for the submicrometre fraction. Particulate matter collected at the two sites was analysed by electron microscopy. The PM from the Acheson process furnace hall is dominated by carbonaceous particles while the samples collected near the pilot furnace are primarily rich in silicon.

## 1. Introduction

Silicon carbide (SiC) is a hard, brittle, ceramic material used primarily for abrasives and cutting tools, but it is also employed in a wide range of other applications, including electronics and diesel exhaust filters. SiC is typically produced in open electrical resistance furnaces, through the so-called Acheson furnace process. In an Acheson furnace, energy is generated by the resistive heating of a graphite core connected to two electrodes at each ends of the furnace. The furnace is built up as the raw materials, a mixture of coke (carbon) and a silica or quartz sand, are placed around the graphite core, as described by Smith et al. [1] and by Smoak et al. in the Encyclopedia of Chemical Technology [2]. The carbon and silica react at high temperatures (1700–2500 °C) to form SiC through a gas phase reaction. The SiC develops as a solid cylindrical block, called ‘the crude’, around the core, with radial layers ranging from graphite as the inner material, over *α*-SiC (the highest grade material with a coarse crystalline, (hexagonal) structure), *β*-SiC, the so-called metallurgical grade SiC, partly reacted raw materials to the unreacted material on the outside. After a cooling period, the crude is separated into its main components and sorted. The product materials are then passed on for secondary processing, such as crushing, milling, washing, drying, sieving, etc. The graphite core, the partly reacted and the unreacted materials are recycled as a new furnace is rebuilt in its place.

The furnaces are operated in cycles and in sets of four, such that four furnaces share one electrical system. At any given time, one of the four furnaces is in the process of being built, one is in active operation, one is cooling down after operation and one is being dismantled. During the building and dismantling of the furnaces, large amounts of raw and product materials are handled with various tools and vehicles. Dust (airborne particulate matter, PM) is thus generated mechanically and dispersed into the air.

During active furnace operation, the thermal and chemical processes in the furnace generate gases and dust [1,3]. The most pressing Health, Safety and Environmental (HSE) concern in the Acheson process furnace hall is the CO gas produced in the process. In modern factories, manual tasks are continuously being replaced by automatic processes and vehicles with vented driver hoods and in this factory, the Acheson process furnace hall remains essentially unmanned for long periods of time. Nonetheless, certain operations, such as maintenance for example, must still be carried out. Hence, personal protective equipment is still essential to protect workers against CO exposure and the staff typically use overpressure facemasks with a pressurized fresh air supply. Anyone entering the furnace building must also wear respiratory protection (dust mask), protective gloves, multiple layers of protective and flame resistive clothing, safety helmet and glasses, etc.

Epidemiological studies have shown that SiC workers have an increased risk of developing lung cancer. Osterman et al. [4] reported that employment in the business of SiC production is associated with an excessive decrement in pulmonary function and that the permissible exposure limits for dust in this industry may not have been adequate to protect workers from developing chronic pulmonary disease. They observed a statistically significant, restrictive pattern of pulmonary function loss related to the duration of work at this SiC production factory. More recent studies by Føreland et al. [3,5] indicate that workers in the SiC industry are exposed to a mixture of agents including quartz, cristobalite, fibrous and non-fibrous SiC and sulphur dioxide. Exposure levels were generally below the current Norwegian occupational exposure limits OELs; however, high exposure to fibres and respirable dust may still occur in the Acheson furnace process area. Johnsen et al. [6] also found a decline in forced expiratory volume (FEV1) (but not the forced vital capacity (FVC)), which was negatively associated with increasing dust exposure, indicating an increased risk of Chronic Obstructive Lung Disease (COPD) in the silicon carbide industry. Romundstad et al. [7,8] called attention to the increased mortality due to asthma, chronic bronchitis and emphysema in the SiC industry and also made a connection between SiC fibre exposure and lung cancer. Skogstad et al. [9] investigated the fibrous and non-fibrous particulate matter in three different Norwegian productions plants and showed that the particle type depends on the plant, process conditions and the raw materials used. There were also significant differences between job functions. Only two sources were identified (1) cleavage particles from crude handling and (2) *β*-SiC fibres from handling of partly reacted material. Many authors, including Gunnæs et al. [10] and Skogestad et al., focus heavily on fibres and seem to suggest that the fibrous particle morphology dominates. This is, however, in direct contradiction to other studies, for example by Bye et al. [11,12], Dufresne et al. [13,14] and Arnoldussen et al. [15], where the fibrous morphologies seem to represent only one out of several morphological categories in the rather diverse palette of the overall particulate matter encountered in a SiC primary production facility.

During recent years, exposure and risk assessment of ultrafine particles (UFPs) has received increasing attention. Control banding is an approach that has been proposed [16], and exposure limits are discussed and suggested [17,18]. UFPs are typically produced during hot processes [19,20,21,22,23,24], and there is no reason to believe that they are not produced in this industry. They are known to be produced in silicon [25], ferrosilicon alloy [26], aluminium production [27] and production and processing of other various ceramic materials [20,21]. None of the above mentioned studies included an investigation of UFP production during SiC production, and nothing is known about the particle size distribution of the submicrometre particles produced.

In order to study the formation of UFPs in the Acheson process furnace hall there are some challenges that must be considered. Different particle sizing equipment exists, but the level of suitability differs between them. The ELPI (Electrical Low Pressure Impactor) instrument by Dekati is often used in rough dusty atmospheres, and the instrument is well known for resilience, a large measurement range and the possibility of collecting size-fractionated samples of the PM for subsequent analysis by, for example, electron microscopy. The real challenge, however, is the size distribution which has significantly lower resolution for the ELPI than the FMPS (Fast Mobility Particle Sizer). The FMPS on the other hand is not as robust as the ELPI is in the most demanding industrial surroundings. Both instruments are large and power consuming. Smaller condensation particle counters (CPC) are available commercially, such as the portable CPC 3007 from TSI, but this instrument only measures concentrations below 100,000 particles/cm^3^, which may be below the industrial pollution level, as it is for the silicon alloy industry [15].

The current study aims to elucidate whether the particulate matter generated inside the hot Acheson furnace during active operation is representative of the overall PM in the furnace hall, and whether the Acheson furnaces are the main sources of UFPs in primary SiC production.

## 2. Materials and Methods

### 2.1. Design

Particle production was studied in two different locations with different furnace configurations. The sampling and measurement equipment were set up next to a small, pilot scale, Acheson furnace and in the main factory hall, where several industrial scale Acheson furnaces were operating simultaneously. It was not a trivial task to find a suitable location for the equipment the main factory furnace hall, as the access to the furnace hall is heavily restricted for safety reasons. In the main factory furnace hall, several other PM generating processes take place which include the handling and transport of raw materials, partly reacted materials and different product materials (crude). This mechanically generated PM is typically coarser than the thermally generated PM and is expected to settle more easily on floors and other surfaces, from which it may become redispersed into the air by vibration or movement. The ELPI was placed in a walking zone, approximately 0.5 m above floor level near one of the furnaces in active operation. There was also a conveyor belt for raw material transport nearby. In the following, the two locations are named the ‘pilot furnace hall’ and the ‘Acheson process furnace hall’.

All persons entering the Acheson process furnace hall must wear a dust mask, protective helmet and glasses, and are provided with a portable personal CO monitoring equipment with an alarm. The staff working for longer periods in the Acheson process furnace hall use overpressure masks with a pressurized fresh air supply. The ventilation system is designed to control the air flow so that fresh air is continuously supplied from a central inlet, close to which are the main walk ways. This also creates an overpressure in the building, which supports the natural convection from the furnaces and steers the overall air flow up towards the roof opening which stretches the length of the factory building.

The pilot furnace was in a more controlled environment as it was placed in a smaller room with a suction hood placed just above the furnace. Here too, anyone entering had to wear the same protective equipment as in the Acheson process furnace hall. During the measurements, however, the external fan was turned off in an attempt to simulate the environment near and above the active furnaces in the furnace hall. The instruments could be placed much closer to the Acheson process and on a level closer to the open pit of the furnace, so that off-gases and dust generated in the furnace process could be more directly collected.

The pilot furnace was built for these experiments; one worker was operating the furnace from an operator’s cabin, separated from the furnace by a wall with windows. The work tasks commonly performed in the Acheson process furnace hall are described by Føreland et al. [3,5]. It was desirable to measure particle size distribution simultaneously with three different instruments, ELPI; FMPS and CPC and the instruments were placed on a table (ca. 1 m above ground) near each other and approximately 3 m from the open top pilot furnace.

### 2.2. Particle Measurement Equipment

#### 2.2.1. FMPS

A Fast Mobility Particle Sizer 3091 (FMPS^TM^) Spectrometer (TSI, Shoreview, MN, USA) was used to measure the particle number concentration and the particle size distribution in the size range 5.6−560 nm. The FMPS has a resolution in 32 channels (16 channels per decade). At the inlet of the FMPS a 1 µm cyclone was installed. The flowrate through the instrument was 10 l/min. Measurements were performed with a time resolution of one second.

The measurement principle of the FMPS is described elsewhere [25]. A dilutor (DIL 550, TOPAS GmbH, Dresden, Germany) was attached to the inlet of the FPMS instrument with a dilution rate 100:1, this was done in order to avoid build-up of dust on the charger needles of the FMPS. The measurements were performed by use of a 0.59 m long flexible conductive silicone tube.

#### 2.2.2. Handheld CPC

A Condensation Particle Counter (CPC, model 3007, TSI, Shoreview, MN, USA) was used in the study. The CPC detects particles in the range of 10 nm–1 µm. The maximum concentration of the instrument is 1 × 10^5^ particles/cm^3^ and the measurement accuracy is ±20% according to the manufacturer. The measurement principle is described elsewhere [25].

The instrument was equipped with a 0.70 m tubing in order to take samples using all instruments at the same place. The results were logged with an averaging period of 1 min for the CPC measurements. Each measurement period lasted a maximum of 2 h before the CPC was loaded with isopropanol alcohol and restarted.

#### 2.2.3. ELPI

A ELPI (model 972E, Dekati Ltd., Tampere, Finland) was used for classifying aerosols according to their aerodynamic diameter, D_p_ and for collection of size-fractionated samples on aluminium substrates. Real-time particle size and concentration measurements in the size range of 7 nm–10 µm was performed through 12 electrometer channels. For the ELPI instruments, each of these channels correspond to one of the substrate “stages”. These stages and their aerodynamic diameter intervals with corresponding geometric mean diameter (GMAD) values are shown in Table 1. These values are used as based for presentations of the particle size distribution presented in the Results section.

A 0.56 m long, flexible tube was attached to the aerosol inlet. Measurements were performed with a time resolution of one second. The measurement principle is described by Kero and Jørgensen [25] and more detailed description of the ELPI function and its principles of operation can be found elsewhere in the literature [28,29,30,31,32].

The aerosols analysed in this study were collected on greased aluminium foil substrates and small pieces of these substrates were cut out and inserted in the scanning electron microscope for analysis. In addition, some material was transferred from substrates onto holey carbon copper grids for transmission electron microscopy.

### 2.3. Workplace Sampling

A particle measurement table was used to house particle monitoring equipment and accessories. In the pilot furnace hall, the table was placed in front of the furnace and the layout was considering equal distance between furnace and all instruments. In the Acheson process furnace hall, the ELPI instrument was placed on a table beside the instruments used by Arnoldussen et al. [15].

### 2.4. Data Analysis

The FMPS measurements are logged by use of the instrument software Fast Mobility Particle Sizer Software version 3.1.1 (TSI, TSI, Shoreview, MN, USA), This software automatically corrects for dilution and other parameters introduced by the applied hardware set-up. Calculation of the UFP number concentration is performed in Microsoft Excel as the sum of the particles from the first 20 channels, resulting in a particle concentration of 5.6–100 nm (named “FMPS-UFP”). The total number concentration within the size range 5.6−560 nm is calculated and named “FMPS”.

The ELPI measurements are logged by the software ELPIvi version 4.0 and Microsoft Excel with a macro supplied by the producer (Dekati Ltd., Tampere, Finland). Only data from size fractions with D_p_ < 0.6 µm are used for comparison with the other two instruments in this study; this concentration range is henceforth named “ELPI”. The UFP number concentration is calculated as the sum of the particles of the three first size bins (7−91 nm) and is named “ELPI-UFP”. In Supplementary Materials, ELPI data for the entire size interval (including the coarse particles) is available. The total number concentration is calculated including all ELPI data and labelled *ELPI_coarse*. In the data treatment for the ELPI, the particles are assumed to have a unit density (1 g/cm^3^).

**Figure 1:**
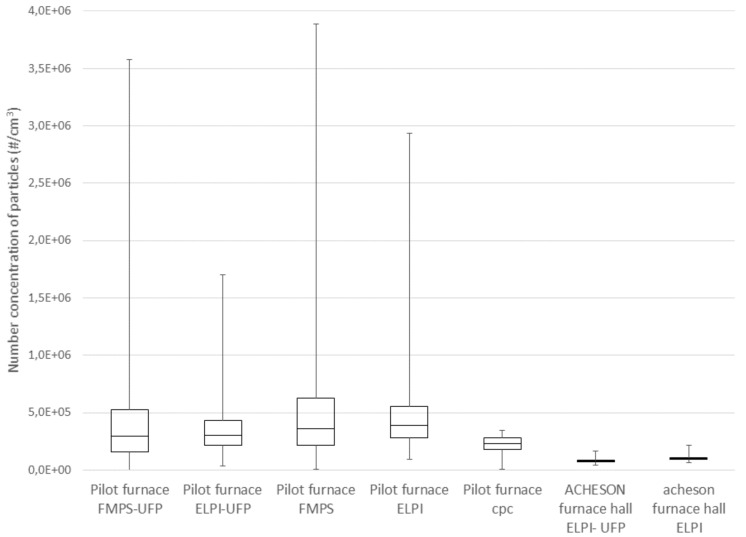
Box-and-whisker plot of the number concentration of particles measured in the pilot furnace hall and in the Acheson process furnace hall. FMPS-UFP: particle concentration of 5.6–100 nm, ELPI-UFP: particles of the three first size bins (7−91 nm), FMPS: Fast Mobility Particle Sizer, ELPI: Electrical Low Pressure Impactor, CPC: Condensation Particle Counter.

Standard measures of central tendency and distributions are calculated as Q1; the 25%, Q2: the median value (50%) Q3: the 75%, minimum value and maximum value. The results are presented in Figure 1. Calculated values for the arithmetic mean (AM), standard deviation (SD), geometric mean (GM), geometric standard deviation (GSD), mean and interquartile range (IQR) are shown as Supplementary Materials (S1).

The data series did not follow a Gaussian distribution (tested by Kolmogorov-Smirnov test, 5%). The correlations between different measures are thus evaluated using the Spearman’s rank correlation coefficient, which is a form of non-parametric statistics and so can be used for non-normally distributed data.

In order to compare the particle size distributions between the ELPI and the FMPS and to render the results comparable to results from other types of instruments, the particle size distribution is reported as a normalised concentration (dN/dlogD_p_):

(1) $$\frac{dN}{d\;logD_{p}}\;=\;\frac{dN}{\log D_{pu}-logD_{pl}}$$

where *dN* is the particle concentration; D_p_ the midpoint particle diameter; D_p*u*_ the upper channel diameter, and D_pl_ the lower channel diameter.

### 2.5. SEM/TEM Analyses

A combination of scanning and transmission electron microscopy (SEM and TEM) was used to study the particulate matter collected by ELPI. The samples are labelled stages 1−12 with increasing particle size. Approximately 50–100 particles were analysed by energy dispersive spectroscopy (EDS) in each sample.

#### 2.5.1. Sample Preparation

SEM: Sample preparation: part of the Al foil (ELPI substrate) was cut out and mounted directly onto a SEM sample holder.

TEM: Sample preparation: Part of the foil (ELPI substrate) was cut out and immersed in isopropanol, which was then put in an ultrasonic bath for 15 min. About 30 drops were dispersed on a 300 M Cu grid with a holey carbon support film. Stages 1, 4 and 6 were selected for TEM analysis.

#### 2.5.2. SEM and TEM Instruments

TEM work was carried out on a JEM-2100F instrument (JEOL, Tokyo, Japan) and the samples were imaged, predominantly in annular dark-field scanning transmission electron microscopy (ADF-STEM mode. The SEM analyses were performed with a dual beam FIB-SEM (FEI Helios Nanolab 600, ThermoFisher, Hillsboro, OR, USA) operated at 15 kV. All images are secondary electron images. All electron microscopy compositional analyses were carried out by EDS.

## 3. Results and Discussion

The particle number concentrations of ultrafine particles and submicrometre particles were measured in the Acheson process furnace hall and in the pilot furnace hall. The statistical results calculated from the measurements are shown in Figure 1 and Table S1.

As shown in Figure 1, the number concentrations of particles are significantly higher near the pilot furnace than in the Acheson process furnace hall, both for the number concentration of UFP and for the submicrometre fractions. The UFP concentration GM was 2.9 × 10^5^ particles/cm^3^ measured by ELPI near the pilot furnace and 7.7 × 10^4^ particles/cm^3^ measured by ELPI in the Acheson process furnace hall. For the submicrometre fractions it was 4.2 × 10^5^ particles/cm^3^ near the pilot furnace and 1.0 × 10^5^ particles/cm^3^ measured in the Acheson process furnace hall, which means that there is a factor of 4 difference between the concentrations in these two halls. This result is counterintuitive as the operators experienced the dust levels in the factory building to be much higher than those near the pilot furnace. Ultrafine particles are generally not visible to the human eye and it is assumed that the experienced high levels of PM in the factory building was primarily due to higher concentrations of coarser PM. The results also show that the maximum concentration near the pilot furnace is up to 4 to 6 times higher than the 75% concentration in the pilot furnace hall, while the concentration in the Acheson process furnace hall is more homogeneous with only a factor of 2 between the 75% and the maximum values. This could be due to the fact that the measurements were performed very close to the source in the pilot furnace hall, with only limited opportunity for mixing with the surrounding air. In the Acheson process furnace hall, the measurements were performed at a greater distance from the source, which gave more opportunity for mixing with the surrounding air and lower peak concentrations.

The concentrations measured were found to be best described by log-normal distributions and were ln-transformed prior to statistical analysis; the data are hence best described by the GM values. The GM of the particle number concentration of UFP in the Acheson process furnace hall was 7.7 × 10^4^ particles/cm^3^ for the UFP fraction and 1.0 × 10^5^ particles/cm^3^ for the submicrometre fraction. No comparable results from the SiC industry are found in the literature. For the UFP concentrations, the concentrations in the Acheson process furnace hall are comparable to concentrations found in the silicon alloy industry using the same type of instruments, measured close to the tapping hole. For the submicrometre fraction, the results from the silicon alloy industry, at 0.7 × 10^5^–0.8 × 10^5^ particles/cm^3^, were nearly identical to the results of the current study while the UFP particle number concentration at 1.9 × 10^5^–2.8 × 10^5^ particles/cm^3^ in a Si plant can be compared to a particle number concentration at 7.6 × 10^4^ particles/cm^3^ in this study [25]. Max peak concentrations were a factor of 2 higher than AM which corresponds to the max peak concentrations found during anode change operations in prebake pot rooms in aluminium smelters, as shown by Thomassen et al. [27]. Compared to other types of industry, the results found in the Acheson process furnace hall in this study seem to be comparable, Evans et al. reported GM values for the number concentration of UFP in a grey iron foundry, ranging from 7.0 × 10^4^–2.8 × 10^5^ particles/cm^3^ [33]. Cheng et al., found AM and GM values of UFP in an iron foundry to be 7.06 × 10^4^ and 6.14 × 10^4^ particles/cm^3^, respectively [34]. Elihn et al., found a comparably high number concentration (1.3 × 10^5^ particles/cm^3^) for aluminium fettling [35]. Kim et al., found GM values in the rubber manufacturing industry, measured by ELPI instrumentation, to be 1.84 × 10^5^ particles/cm^3^ for the entire measuring period, but 5.45 × 10^5^ particles/cm^3^ for the ‘final process’ [36] and Fonseca et al., found exposure concentrations between 1.4 × 10^5^ and 5.3 × 10^5^ particles/cm^3^ in the ceramic tile industry [20].

**Figure 2:**
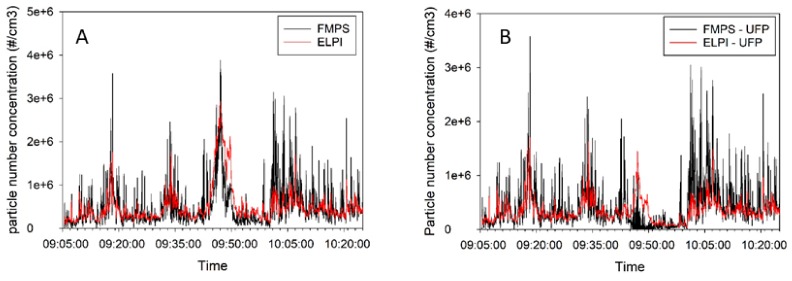

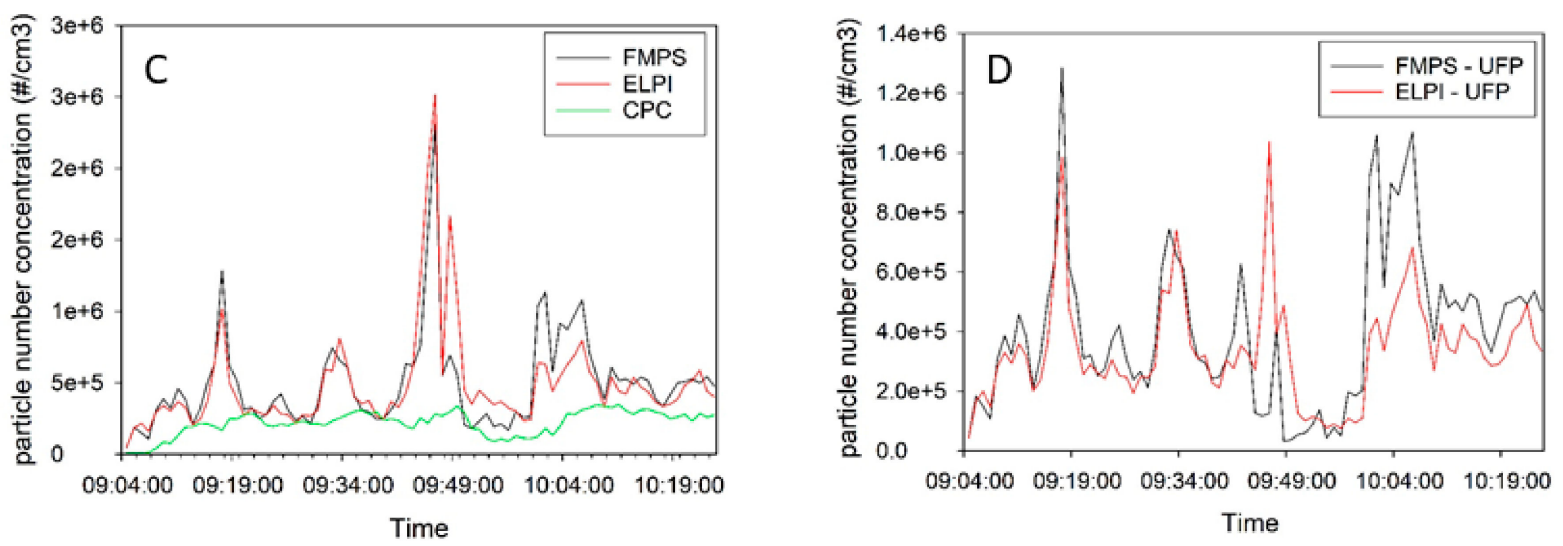
Number concentration of emitted particles from the pilot furnace hall (**A**) the submicrometre fractions measured with ELPI and FMPS; 1 sec. resolution (**B**) ultrafine particles measured with ELPI and FMPS; 1 sec. resolution (**C**) the submicrometre fractions measured with ELPI, FMPS and CPC; 1 min resolution and (**D**) ultrafine particles measured with ELPI and FMPS; 1 min resolution.

Figure 2 shows the particle number concentration measurements as a function of time taken near the pilot furnace with CPC, FMPS and ELPI. Figure 2A,B compare FMPS and ELPI measurements performed with one second resolution, and Figure 2C,D show the measurements with one minute resolution. Figure 2C includes CPC measurements. Figure 2A,C show the submicrometre fractions, while Figure 2B,D show the UFP fractions.

**Figure 3:**
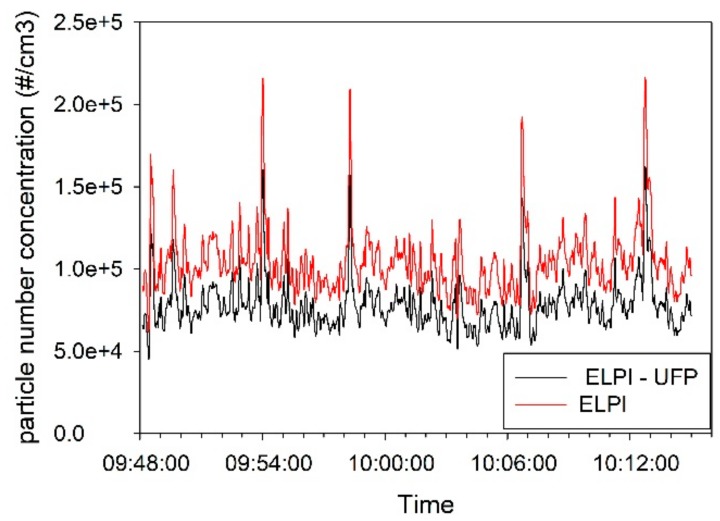
Particle number concentrations (#/cm^3^) as a function of time for measurements in the Acheson process furnace hall.

The concentrations inside the Acheson process furnace hall were only measured by the ELPI instrument due to the dusty and demanding atmosphere. The results of the measurement are shown in Figure 3.

The concentration versus time measurement in the pilot furnace hall (Figure 2) shows a pollution source with some fluctuation during this time period. This is also reflected in large standard deviations in Table S1. No specific reasons for the peaks were identified. The concentration vs. time measurements at the Acheson process furnace hall (Figure 3) also showed fluctuations, but to a substantially smaller extent. As seen in Table S1, the standard deviation is approximately 20% of AM values. No specific reasons for the peaks were identified.

As illustrated in Figure 2 the FMPS measurements agree well with the ELPI measurements and the FMPS-UFP agree well with the ELPI-UFP. The correlations between FMPS and ELPI (Figure 2A) and the correlation between FMPS-UFP and ELPI-UFP (Figure 2B) is evaluated by means of Spearman rank coefficients (*ρ*) and are acceptable with *ρ* = 0.761 for FMPS/ELPI and *ρ* = 0.635 for FMPS-UFP/ELPI UFP based on 1 sec. measurements (N = 4771). The CPC measurements were performed with 1 min resolution, with the result that the FMPS and ELPI results are recalculated to 1 min values for the illustration in Figure 2C,D, the correlation improves by this to *ρ* = 0.848 for FMPS/ELPI (Figure 2C) and *ρ* = 0.773 for FMPS-UFP/ELPI UFP (N = 81). The correlation between the CPC and 1 min calculated values for FMPS and ELPI is unacceptable, with *ρ* = 0.377 for FMPS/CPC and *ρ* = 0.377 for ELPI/CPC. All correlations were significant at the 0.01 level (2-tailed).

**Figure 4:**
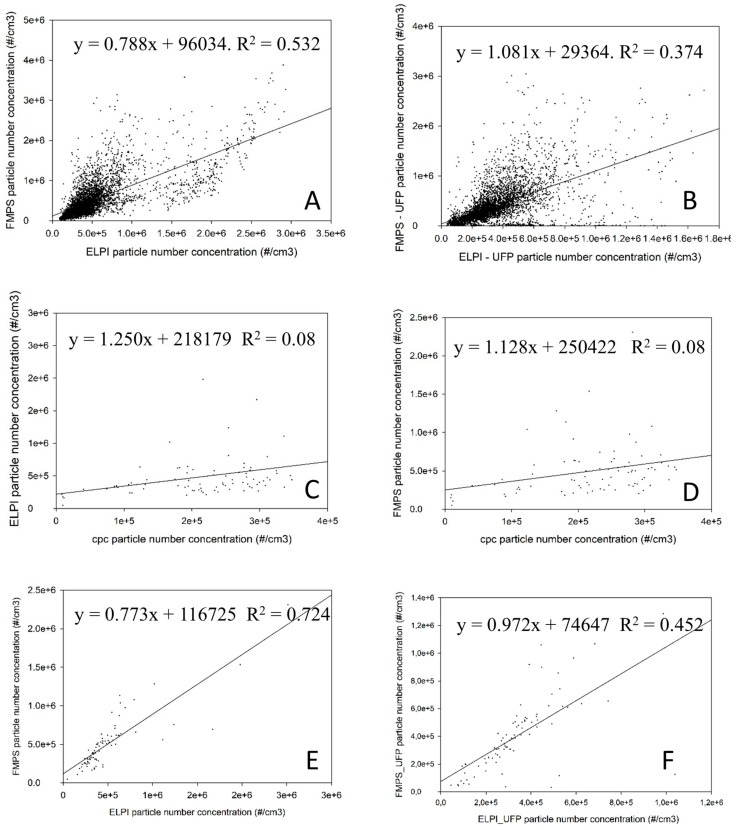
Scatterplot of comparable measurements: (**A**) ELPI compared to FMPS; 1 sec resolution; (**B**) ELPI-UFP compared to FMPS-UFP; 1 sec resolution.; (**C**) CPC compared to ELPI, 1 min resolution.; (**D**) CPC compared to FMPS 1 min resolution.; (**E**) ELPI compared to FMPS; 1 min resolution.; (**F**) ELPI-UFP compared to FMPS-UFP 1 min resolution.

The FMPS measurements seem to show a higher degree of fluctuation, which was also found by Kero and Jørgensen in the silicon alloy industry [25] The CPC concentrations were found to be significantly lower than the ELPI and the FMPS concentrations. The CPC concentration values are almost twice as high as the maximum concentration range recommended by the producer. Consequently, the CPC concentration values are deemed unreliable at this location. In order to make a visual illustration of this, scatterplots is shown in Figure 4. The correlation coefficients (R^2^) as illustrated in Figure 4 supports the results of the spearman rank analyses showing highest correlation coefficients for FMPS/ELPI with measurements recalculated to 1 min values (R^2^ = 0.724), followed by R^2^ = 0.532 for the original 1 s measurements by FMPS/ELPI, R^2^ = 0.452 and R^2^ = 0.374 for FMPS-UFP/ELPI-UFP but only R^2^ = 0.08 for the ELPI/CPC and FMPS/CPC results.

### 3.1. Particle Size Distribution

**Figure 5:**
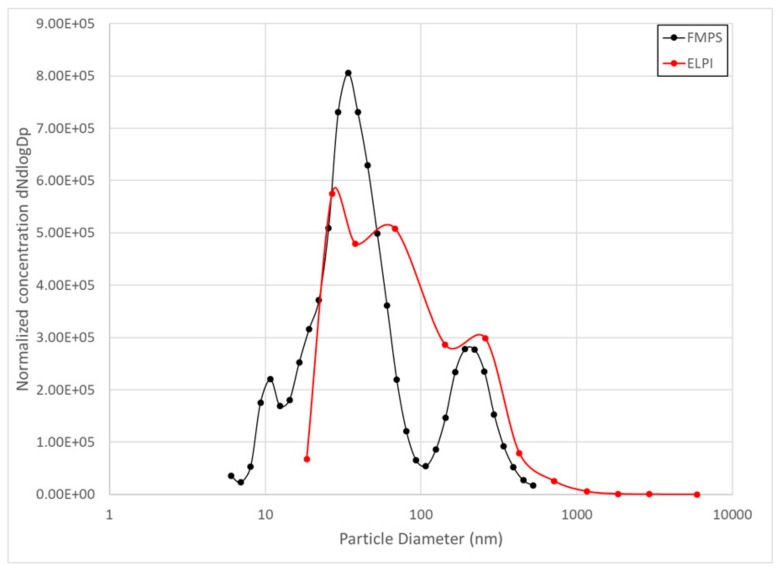
Particle size distribution of the ELPI and the FMPS in the pilot furnace hall.

The particle size distributions of the ELPI and the FMPS from the locations near the pilot furnace are compared in Figure 5.

The particle size distribution measured by the FMPS instrument shows a higher percentage of the particles in the smallest fraction compared to the ELPI instrument for the measurements in the furnace hall. The percentage of particles below 54 nm and 100 nm is shown in Table 2. Near the pilot furnace, 70% of the particles measured by FMPS is below 54 nm and 79% of the particles is below 100 nm, compared to the ELPI measurements where 48% of the particles is below 54 nm and 70% is below 100 nm. This correspond to the results from Leskinen, who found that the FMPS showed a smaller particle size than the SMPS and ELPI [37]. Price et al., confirm this, stating that divergence was generally noted at the lower and upper working size range of the instruments, when comparing ELPI, FMPS, SMPS and APS [38].

**Figure 6:**
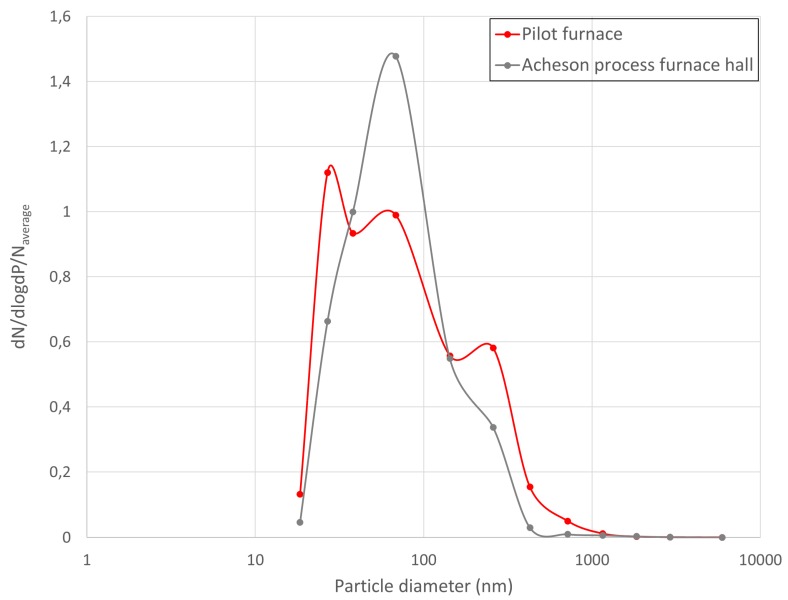
Comparison of particle size distribution in the pilot furnace hall and at the Acheson process furnace hall-data presented as dNdlogDp normalized with respect to total concentration dN/dlogdP/N_average_.

In the Acheson process furnace hall, only ELPI was used for measurements. The average number concentration measured in the pilot hall and the Acheson process furnace hall differ considerably (see Figure 1), and to allow for a graphic comparison between particle size distribution in the pilot hall and at the Acheson process furnace hall, in Figure 6, the data have been normalized to the average mean concentration of the two locations. The particle size distribution shows a slightly higher fraction of particles below 54 nm in the pilot furnace hall compared to the Acheson process furnace hall (48% compared to 41%) but slightly lower percentage of particles in the ultrafine range (70% compared to 77%). As seen from Figure 6 the dominating particle size fraction in the Acheson process furnace hall had a geometric mean aerodynamic diameter of 68.23 nm.

The difference in particle size distribution between the Acheson process furnace hall and the pilot furnace hall may have several reasons. First, the instruments were placed closer to the pilot furnace than to the furnaces in the Acheson process furnace hall and therefore measure the emission more directly from the active furnace. Hence, the pilot furnace hall results vary more and are more sensitive to furnace function. Another difference is that in the pilot furnace hall, only one active furnace was operated, and there were no simultaneous processes in the room. That is to say, no furnaces were dismantled, under construction or cooling, no vehicles were moving, no other operations were carried out in the hall. While in an industrial scale Acheson furnace hall, several furnaces are operating simultaneously, and many different processes and operations always take place simultaneously. The Acheson process furnace hall is very large, and the measurements will inevitably be influenced by transport of particles from other processes in the hall, like material handling and transport, incomplete combustion or other thermal processes, as well as dilution of the emissions or possible particle agglomeration and coagulation processes.

In order to compare the concentrations measured by the ELPI instrument with the concentration measured by FMPS instrument, the ELPI results is calculated for the stages below 600 nm. The coarse fraction of the number concentration of the particles, as measured by the ELPI (stages 8–12) does not contribute to the results compared to the ELPI fraction as presented in this study. The statistics is nearly identical for the ELPI and the ELPI_coarse fraction (Table S1), and the concentration versus time curves is showing exactly the same fluctuations (Figure S1), which also is reflected with regression coefficients R^2^ = 0.999 at the pilot furnace hall and R^2^ = 1.000 at the Acheson process furnace hall (Figure S2). This means that the particle emission of airborne particulate matter from a primary SiC production plant are dominated of particles below 600 nm, when the particles emission is evaluated as number concentrations of the particles.

### 3.2. Electron Microscopy Results

During TEM and SEM analysis, 50–100 particles were analysed by EDS in each sample. There was, however, clear differences in composition between particles of different morphology (shape) which means that after a first mapping of constituents had been performed it was possible to make fast visual assessments of more samples, without the use of EDS. Thus, the statistical basis is, in practice, much larger than the number of particles analysed by EDS.

SEM results for samples from ELPI stage 5 and smaller (both sampling locations): secondary electron images give no or little information. The particles are totally embedded in the grease and cannot be seen. The electron beam burns off the grease after extended electron beam exposure, after which we can observe some small particles at high resolution. These ultrafine particles appear to be spherical in shape and look much like amorphous silica but the presence of the grease made it impossible to draw any firm conclusions about their chemical composition or agglomeration status.

**Figure 7:**
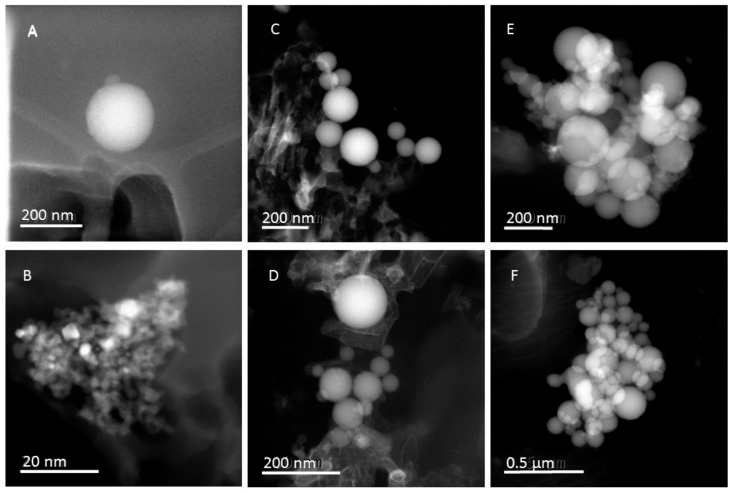
TEM (transmission electron microscopy) micrographs of ELPI samples. Micrographs (**A**,**B**) are from the pilot furnace, ELPI stage 2, illustrating silica spheres and agglomerated metallic nanoparticles, respectively. The silica spheres in micrograph (**C**,**D**) are from the pilot furnace hall, ELPI stage 4 and the silica spheres in micrographs (**E**,**F**) are from the Acheson process furnace hall, ELPI stage 6.

Samples from stages 4–6 (both sampling locations) were then examined by transmission electron microscope observation but sample preparation proved challenging and the particles documented by TEM may not be entirely representative. Nonetheless, the presence of ultrafine, spherical, amorphous silica particles (mostly agglomerated) could be confirmed, see Figure 7. The formation of amorphous silica particles, including ultrafine particles, from SiO gas in thermal processes is well known in the literature and this type of particle is relatively common in primary silicon production [25,39,40]. From TEM assessment, the sphere diameters in stages 4 and 6 were approximately 20−90 nm and 40−300 nm, respectively.

Other ultrafine particles identified in the TEM samples from the Acheson process furnace hall were carbon/organic UFPs which appeared to be crystalline but disintegrated under the electron beam so that they could not be studied in any detail. In samples collected near the pilot furnace, on the other hand, there were some instances of agglomerated, metallic ultrafine particles, see Figure 7B. The metallic components were Cu, Pt and Sn but the Cu signal may be from the copper-carbon-grid sample holder.

SEM results for samples from ELPI stage 7 and higher, collected near the pilot furnace were dominated by the following crystalline phases in decreasing order of frequency: Si, C, SiO_2_, and SiC. Chemical composition analysis by EDS detected Fe, S, K and Na impurities, some of which may have been added with the grease. In the samples collected in stages 9–11, there are both fibrous and non-fibrous SiC particles. In samples collected in the ELPI stage 7, only very few SiC particles were detected but the ones found were all in the form of fibres or whiskers.

**Figure 8:**
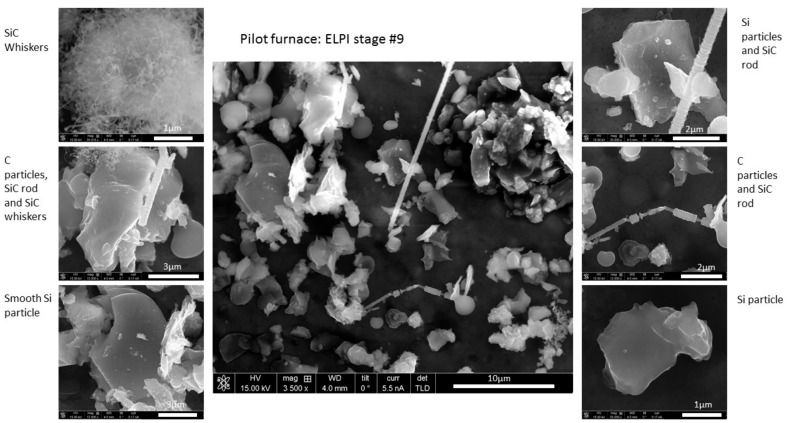
SEM results showing the particle types and morphologies collected on stage 9 in the ELPI near the pilot furnace.

**Figure 9:**
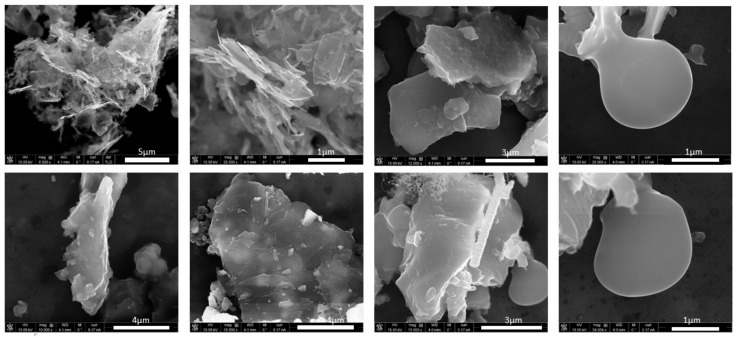
SEM results illustrating the different morphologies of carbon-rich particles near the pilot furnace found in stages 9 and 10.

Figure 8 shows the various particle types and morphologies collected at stage 9 in the ELPI near the pilot furnace and Figure 9 shows the different morphologies of the carbon particles collected in stages 9 and 10 of the ELPI near the pilot furnace. The particle shapes and compositions are in agreement with previously published articles by Bye et al. [12]. Arnoldussen et al., collected their samples at the same location as the ELPI in the Acheson process furnace hall [15]. They used a filter set up which collected PM during the same period as the ELPI but for a much longer time period (without the size limitations of the ELPI). Hence, the particle size and compositions seem to differ somewhat, but our results confirm their overall picture of a very diverse PM situation.

SEM results for samples from ELPI stage 7 and higher, collected in the Acheson process furnace hall contained the following crystalline phases in decreasing order of frequency: C, SiO_2_, Si and SiC. Chemical composition analysis by EDS detected Fe, S, Ca and Na impurities but, again, some of the impurities may be from the grease. The strongest signal in the EDS spectra was actually Al but this signal is assumed to come from the Al foil and is therefore not discussed. Arnoldussen et al., found no Fe or S but significant amounts of Al, Ti and V (Ti and V are common alloying elements for Al-alloys) [15].

Quantitative carbon analysis by EDS is highly uncertain as C is a relatively light element and for the TEM analysis, there is also C in the sample holder. The conclusions herein are consequently of a qualitative nature and are primarily based on SEM results. Nonetheless, C appears to be the major constituent of the largest class of particles observed here. The carbonaceous particles are likely to be mechanically generated airborne particulate matter from the raw materials (coke) which may have become dispersed in the air whilst in conveyor belt transport close to the sampling site. However, carbonaceous particles may also have other sources, for example soot from incomplete combustion from the furnace process. For samples collected on the ELPI stages 10 and higher, SiC particles are present but only as a minority phase and the phase composition is heavily dominated by C-rich particles.

### 3.3. Comparison with Nano Reference Values

No occupational exposure limits (OELs) are available regarding nanoparticles or nanomaterials yet. Despite this, exposure limits proposed for nanoparticles exists, regarding worker exposure to engineered nanoparticles [16,17,18] (mentioned nano reference values (NRV)). The NRV values relate to the density of the particles, to the form and to biopersistency. Class 1 hold nanofibers with a NRV value at 0.01 fibre/cm^3^. Class 2 hold nanomaterials with density is below 6000 kg/m^3^ the NRV is 40,000 particles/cm^3^ given as 8 h TWA value, and class 3 includes nanomaterials with a density of 6000 kg/m^3^, the NRV is 20,000 particles/cm^3^. The last class (4) involve non-biopersistent granular nanomaterials where the NRV value is the applicable OEL [17]. It has been discussed if SiC was non-biopersistent, but newer toxicological studies of SiC- nanoparticles have questioned this, showing that SiC-NPs are accumulated in lung cells, in vitro, where they do not cause strong cell mortality, but induce major redox disturbance and DNA damage. These cellular responses both depend on SiC-NP cluster size, and on Si/C ratio [41]. The structure of the NRV values shows that non-biopersistent nanoparticles with low density hold the highest risk. The density of SiC is 3210 kg/m^3^, which means that the NRV for SiC particles is 20,000 particles /cm^3^.

The concentrations found in this study relates to working area concentrations in the furnace halls and is not performed as worker exposure measurements in the breathing zone. This means that the results are not directly comparable with the NRV values. Measurements in working area’s is normally said to underestimate worker exposure, but this depend on the nature of the work, the nature of the process and if the workers are using personal protection equipment. The work area concentrations found exceed the NRV values, in the pilot furnace hall and in the Acheson process furnace hall. In this company, however, all workers wear dust masks, even just passing through the furnace hall. Workers entering this area regularly are using supplied air respirators and most workers actually perform their tasks from a ventilated vehicle cabin. The company frequently follow up with respirator fit testing of all workers.

### 3.4. Limitations

The use of silicon tubing could cause a risk of particle deposition in the tube as pointed out by Tsai [42] and Kumar et al. [43]. Ideally, particle deposition in the tube should be taken into account, but experimental studies have difficulties in determining how large the particle loss is, and for which particle sizes. Tsai report that there is a maximum of 30% loss for particles with the size 8 nm by using a 8.4 m tubing, and that low particle loss was observed for particles greater than 40 nm [42], while Kumar et al., report modest particle losses for particles below 20 nm [43].

Particle diffusion losses could, in principle, influence the measurements performed by two different instruments. The FMPS instrument however is developed with an inversion matrix regarding this aspect, which means that diffusion losses are considered in the matrix. The situation is similar for the ELPI where correction algorithms are included in the operation software. These corrections are thoroughly described in the literature [28,30,31,32].

No recalculations are performed according to the particle deposition in the tubing, nor diffusion losses in the instruments in this study, since recalculation already is done within the FMPS and ELPI instruments, and might introduce an undesired risk of introduction of a source of error regarding the relatively short tubing’s used. In order to keep the particle deposition as low as possible, the tubing was kept as straight as possible, and the bends as gradual as possible.

The measurement accuracy between the instruments compared differ. The manufacturer of the CPC instrument gives the uncertainty of the instrument, as a percentage of the concentration measured. Corresponding values does not exist for the ELPI or the FMPS instruments. Uncertainty of the measurement relates to both instrument and to factors like measurement procedure, placing of the instruments, use of tubing’s, number of measurement etc. In general, instrument comparisons made in real industrial settings is difficult, it is however important to investigate the differences between measurement equipment in real industrial settings in order to make recommendations for measurements of ultrafine particles.

## 4. Conclusions

Three different instruments for concentration and size assessment of airborne particulate matter were used near a pilot scale Acheson furnace during active operation. The FMPS and the ELPI showed acceptable correlation of data at this location and their measured number concentrations of submicrometre and ultrafine particles, as well as their respective particle size distributions, are documented here. The FMPS seem to classify a larger fraction of the detected particles as ultrafine than the ELPI does. This effect may be a consequence of the superior size resolution of the FMPS particle size distribution.

The CPC concentration values were out of range and are deemed unreliable. The use of the CPC cannot be recommended for this type of location and process.

The ELPI, being the most robust of the three instruments, was also applied inside the main factory building, in the Acheson process furnace hall. The concentrations in the industrial Acheson process furnace hall was 7.7 × 10^4^ particles/cm^3^ for the UFP fraction and 1 × 10^5^ particles/cm^3^ for the submicrometre fraction. This is comparable to the concentrations measured in other industries.

Particulate matter collected at the two sites was analysed by electron microscopy. The PM from the Acheson process furnace hall is dominated by carbonaceous particles while the samples collected near the pilot furnace are primarily rich in silicon. Thus, the particulate matter generated through thermal processes inside the Acheson furnace is not representative of the overall PM in the furnace hall where several types of sources contribute to the overall PM.

## Figures and Tables

**Table 1:** Aerodynamic diameter intervals (D_p_) and geometric mean aerodynamic diameters (GMAD).

Stage No	D_p_ Range (µm)	GMAD (µm)
1	0.007–0.028	0.02
2	0.028–0.054	0.04
3	0.054–0.091	0.07
4	0.091–0.153	0.12
5	0.153–0.259	0.2
6	0.259–0.379	0.31
7	0.379–0.609	0.48
8	0.609–0.942	0.76
9	0.942–1.59	1.22
10	1.59–2.38	1.95
11	2.38–3.97	3.07
12	3.97–9.85	6.25

**Table 2:** Percentage of particles in the ultrafine range and below 54 nm.

	Percentage UFP	Percentage <54 nm
	(dNdlogDp) (%)	(dNdlogDp) (%)
ELPI Acheson process furnace hall	77	41
ELPI Pilot furnace hall	70	48
FMPS Pilot furnace hall	79	70

UFP: ultrafine particles.

## Supplementary Materials

The following are available online at www.mdpi.com/1660-4601/14/12/1611/s1, Figure S1: Particle number concentrations (#/cm^3^) as a function of time for the fractions ELPI_UFP, ELPI and ELPI_coarse in (A) the pilot furnace hall and (B) the Acheson process furnace hall, Figure S2: Scatterplot of (A) ELPI compared to ELPI coarse—pilot furnace hall, (B) ELPI compared to ELPI coarse—Acheson process furnace hall, Table S1: Statistics for the different measurements performed divided by furnace type and instrument used.
