# Supplementary material for: Real-Time Measurements and Characterization of Airborne Particulate Matter from a Primary Silicon Carbide Production Plant
Source: Int J Environ Res Public Health. 2017 Dec 20;14(12):1611. doi: 10.3390/ijerph14121611 (PMC5751027; doi:10.3390/ijerph14121611)
Supplement: Supplementary file 1 [file ijerph-14-01611-s001.pdf]

Supplementary Materials

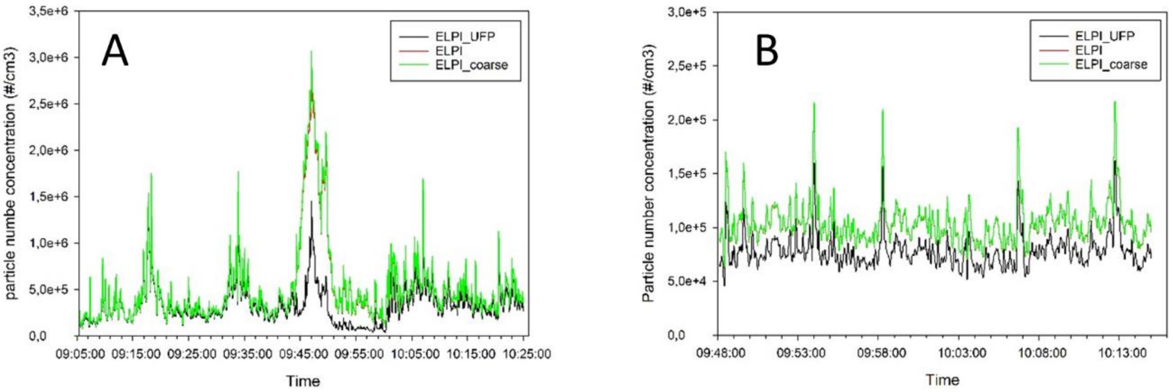

Figure S1. Particle number concentrations (#/cm^3) as a function of time for the fractions ELPI_UFP (Electrical Low Pressure Impactor_ultrafine particles), ELPI (Electrical Low Pressure Impactor) and ELPI_coarse in (**A**) the pilot furnace hall and (**B**) the Acheson process furnace hall.

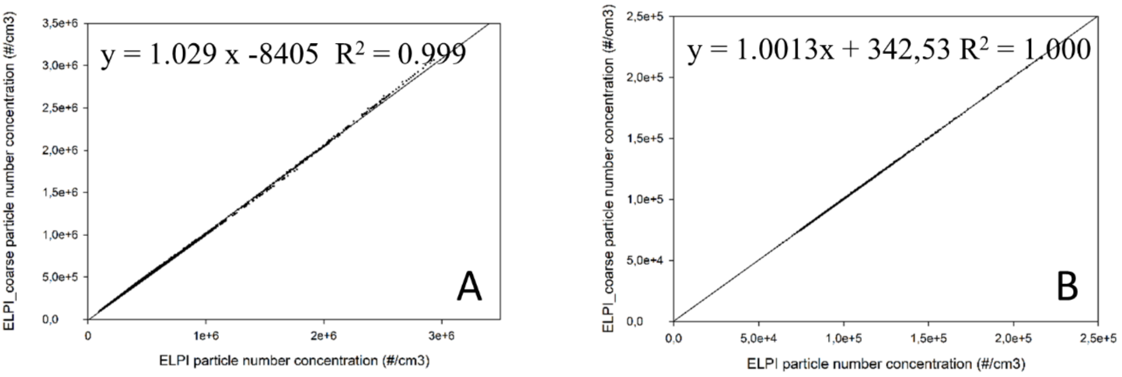

Figure S2. Scatterplot of (**A**) ELPI (Electrical Low Pressure Impactor) compared to ELPI coarse – pilot furnace hall, (**B**) ELPI compared to ELPI coarse – Acheson process furnace hall.

Table S1. Statistics for the different measurements performed divided by furnace type and instrument used.

Location		AM [1]	SD [2]	GM [3]	GSD [4]	Median	Min [5]	Max [6]	IQR [7]	N [8]
The Acheson process furnace hall	ELPI_UFP	77,993	14,974	76,781	1.19	75,713	45,576	162,348	13,957	1651
The Acheson process furnace hall	ELPI	104,505	19,849	102,926	1.18	101,287	62,001	216,545	17,987	1651
The Acheson process furnace hall	ELPI_coarse	104,990	19,877	103,414	1.18	101,808	62,705	217,253	18,030	1651
The pilot furnace	FMPS_UFP	404,906	390,089	245,641	3.38	295,356	13	3,579,381	361,730	4771
The pilot furnace	ELPI_UFP	344,201	212,633	287,580	1.87	300,649	36,936	1,702,085	220,317	4771
The pilot furnace	FMPS	507,801	462,333	369,490	2.22	358,616	5,040	3,883,427	412,230	4771
The pilot furnace	ELPI	513,091	425,934	415,889	1.82	389,782	90,319	2,933,451	274,613	4771
The pilot furnace	ELPI_coarse	519,690	438,468	419,810	1.82	393,765	90,499	3,068,563	276,953	4771
The pilot furnace	CPC	218,082	85,656	183,656	2.18	233,431	8844	347,347	102,442	81

[1] arithmetic mean (AM), [2] standard deviation (SD), [3] geometric mean (GM), [4] geometric standard deviation (GSD), [5] minimum, [6] maximum, [7] interquartile range (IQR), [8] number of measurements (N).
